# Giant Pyonephrosis Diagnosed Using POCUS in a Resource-Limited Setting

**DOI:** 10.24908/pocusj.v10i02.19112

**Published:** 2025-11-17

**Authors:** Roody Menager, Rebecca St Louis, Anst Gelin, Flawendjee Djaweelentz Jacques

**Affiliations:** 1 Department of Emergency Medicine, Hôpital Universitaire de Mirebalais, Mirebalais, Department du Centre, HTI; 2 Department of Emergency Medicine, Hennepin County Medical Center, Minneapolis, Minnesota, USA; 3 University of Minnesota Medical School, Minneapolis, Minnesota, USA

**Keywords:** POCUS, Giant pyonephrosis, Infected stone, Obstructed ureter, Percutaneous nephrostomy, Ultrasound guided aspiration

## Abstract

Pyonephrosis is a severe complication of hydronephrosis and can lead to the destruction of the renal parenchyma, sepsis, shock, and death. The clinical presentation is nonspecific, and 15% of patients are asymptomatic on presentation. In resource-limited settings, where Computed Tomography (CT) imaging is not available, point of care ultrasound (POCUS) plays an important role in evaluating kidney diseases in the emergency department (ED). In this case, a 36-year-old man presented to the ED with dyspnea, fever, abdominal pain, and abdominal distention in the area where an intra-abdominal tumor was reported. After ultrasound-guided aspiration, pyonephrosis was diagnosed. In the absence of other capable specialists, the emergency physician performed a percutaneous nephrostomy procedure, and eight liters of pus were removed. The patient improved significantly and was discharged from the ED with oral antibiotics and scheduled urology follow-up in the clinic.

## Introduction

Pyonephrosis is a severe complication of hydronephrosis, which is defined as the accumulation of pus at the pyelocaliceal cavity-level. While it is a rare condition, early diagnosis and treatment are paramount given that pyonephrosis can progress to sepsis, with a mortality of 20% to 42% [1]. Even when the patient survives, it often leads to complete destruction of the renal parenchyma, which renders the kidney non-functional [2].

Pus formation is generally seen in moderate or severe hydronephrosis. Although the cause of obstruction varies, a left kidney preference [3] and a slight female predominance are reported (56.5% versus 43.5%) [4]. From a radiology standpoint, a giant pyonephrosis is defined as a purulent collection that is greater than 1000 cc in volume, or crosses the midline of the abdomen, or is measured at the height of 5 vertebral bodies [5].

Computed Tomography (CT) imaging or aspiration of pus are the gold standards for diagnosing pyonephrosis. In addition to identifying pyonephrosis, CT imaging can provide additional information such as the quality and quantity of pus present, the degree of hydronephrosis, the presence of an abdominal mass as the cause of the obstruction, and the location and size of an obstructing stone. However, CT imaging is not a realistic option in resource-limited settings due to its low availability. Instead, point of care ultrasound (POCUS) and diagnostic ultrasound are more readily accessible and remain important tools for the assessment of pyonephrosis with good sensitivity and specificity (90% and 97%, respectively) [6].

This case presentation explores POCUS diagnostic techniques and special therapeutic interventions for a patient with massive pyonephrosis in a resource-limited setting.

## Case Description

A 36-year-old man with no known medical history was seen in the Emergency Department (ED) at the University Hospital in Mirebalais, Haiti, for fever and abdominal pain. The patient reported a prolonged fever lasting multiple weeks, followed by generalized abdominal pain and a slight increase in the size of his abdomen. Despite multiple visits to other institutions where he received recurrent doses of analgesics, the patient's symptoms continued to worsen.

On arrival to the ED, the following vital signs were recorded: blood pressure 118/74 mmHg; heart rate 146 beats per minute; respiratory rate 28 cycles per minute; temperature 38°C; and blood oxygen level 100% on room air. On physical examination the patient was noted to be drooling and tachypneic and had a dry cough. He was alert and oriented to person, place, and time. He had pale palpebral conjunctival and scleral icterus. His breath sounds were slightly decreased at the right lung base. His abdomen was mildly distended with a firm, painful lump extending from the left flank to the left lower quadrant/pelvis.

Cardiac POCUS examination was normal, as was the right kidney. The left kidney was not visualized, and there was a cystic mass in the left upper quadrant extended to the pelvis. At this point, the patient was assumed to be septic, likely due to pneumonia with or without COVID-19. An intra-abdominal neoplastic process was also suspected. He underwent fluid resuscitation with Lactated Ringer's solution and was given antibiotics (ceftriaxone and doxycycline) and analgesics (morphine and acetaminophen).

**Figure 1.**
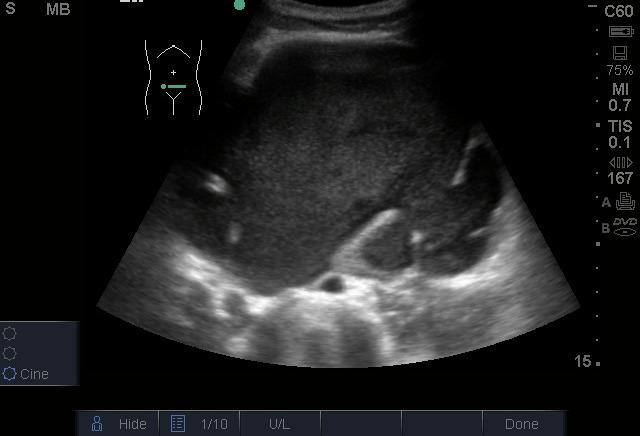
Cystic lesion extending through the pelvic area.

**Figure 2.**
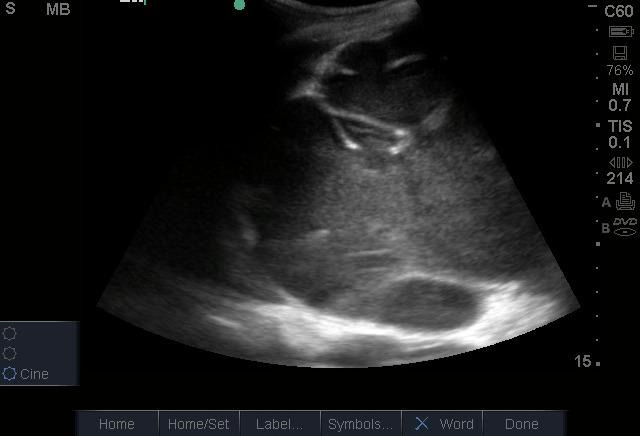
Hypoechoic content in the left upper quadrant with thick incomplete septa.

**Figure 3.**
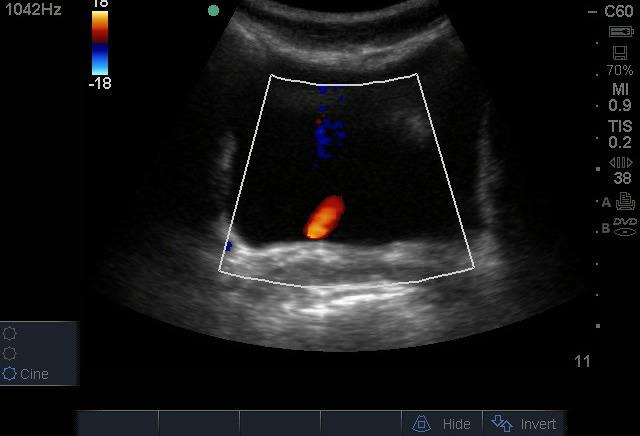
Visualization of a normal right-ureteral jet and no evidence of a left-side jet.

On his first day in the ED, his laboratory evaluation returned with a white blood cell (WBC) count of 22.5 X 103 cells/µL with a 78.7% neutrophil predominance, a hemoglobin of 4.5 g/dL, and creatinine of 1.64 mg/dl. On ED day 2, the patient became more toxic in appearance with persistent abdominal pain. His WBC count increased to 29.2 X 103 cells/µL with 84.3% neutrophils. At that point, POCUS was repeated by an emergency physician with advanced training in POCUS. This exam showed the absence of a left kidney and the presence of a large complex hypoechoic cystic mass that fully occupied the left side of the abdomen extending to the pelvis (Figures 1, 2). The mass contained poorly vascularized, thick, incomplete septations and layering fluid levels. The bladder was evaluated for ureteral jets which were absent on the left side (Figure 3), suggesting a complete obstruction of the left-side collecting system. It was then recognized that the mass was an obstructed left kidney, and the poorly vascularized septations were remnants of the renal parenchyma. Without the availability of interventional radiology in the country or urgent nephrology/urology in the hospital, the emergency physician used ultrasound guidance to aspirate purulent fluid from the left kidney. This was followed by a percutaneous nephrostomy tube placement.

**Figure 4.**
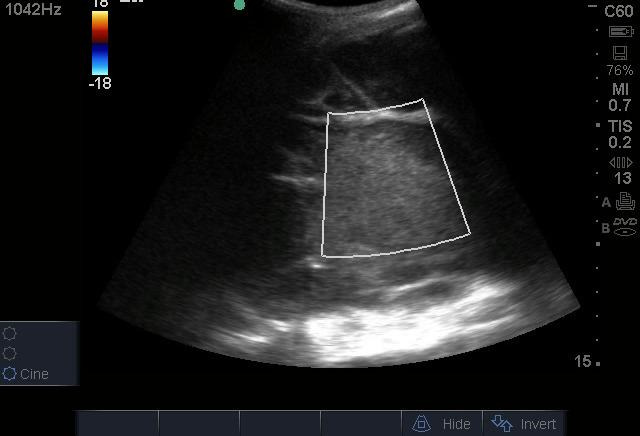
No evidence of color Doppler flow during the left flank assessment.

**Figure 5.**
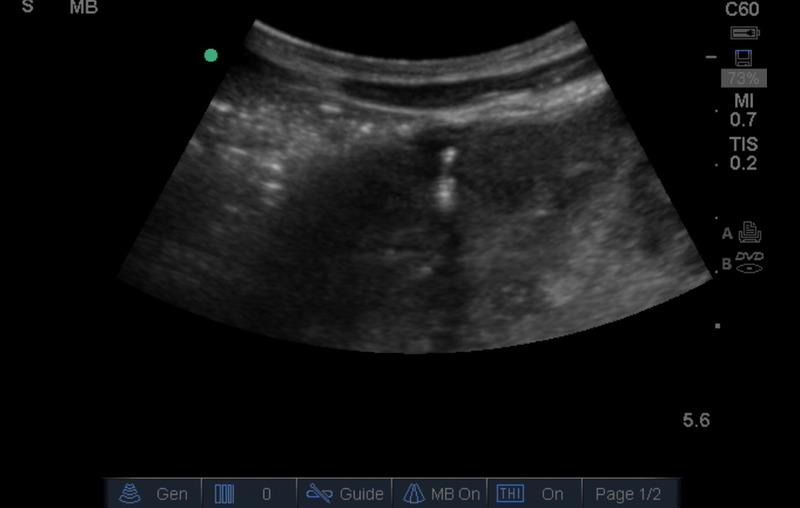
Ultrasound-guided pigtail placement in the left flank.

**Figure 6.**
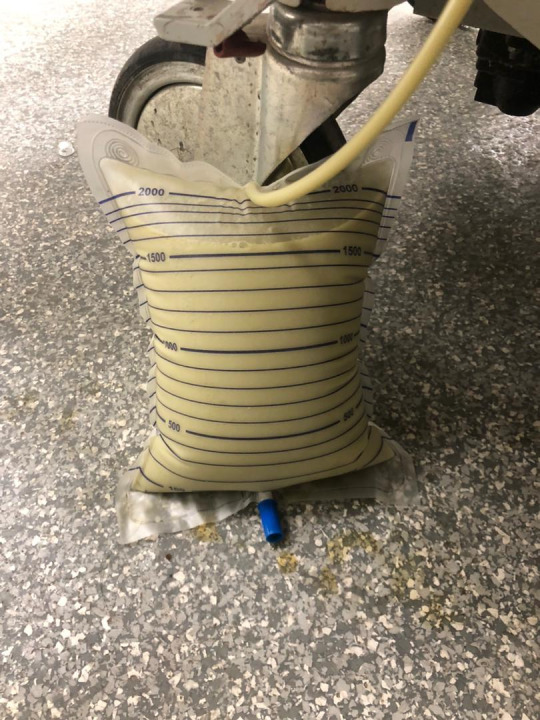
Pus drained during the percutaneous nephrostomy (eight liters).

The patient was first placed in the right lateral decubitus position and the area was inspected for procedural planning using a Sonosite Micromaxx ultrasound machine with a 2-5 MHz curvilinear probe (FUJIFILM Sonosite, Washington, United States). After finding an appropriate location, Doppler was used to confirm the absence of vascular structures (Figure 4) and the skin was anesthetized with a small amount of 1% lidocaine using a 23-gauge needle. An 18-gauge needle was used to enter the retroperitoneal space using sterile techniques; the needle tip was always visible under ultrasound guidance (Figure 5). After confirming with aspiration, an 8-French pigtail catheter was inserted using Seldinger techniques. The pigtail catheter was secured to the skin with non-absorbable sutures. Because a commercial drain was not readily available, intravenous catheter tubing was fitted to the end of the pigtail catheter. The reservoir was removed from the other end of the tubing, and it was inserted and taped into a Foley bag. Normal flow was noted on this improvised container (Figure 6).

The patient was monitored closely for complications. The culture grew Escherichia coli that was sensitive to cefepime and imipenem. In addition to the removal of 8, 000 ml of purulent fluid by the emergency physician under ultrasound guidance, adjustments were made to the antibiotic regimen based on the resulted culture. On ED day 7, the patient's WBC count normalized to 11.3 X 103 cells/µL, and his clinical status improved. The patient was discharged from the ED with the pigtail catheter in place and advised to urgent outpatient follow-up with urology (within 1-2 weeks), at the next time the urologist was in town.

## Discussion

Pyonephrosis has similar presentations to pyelonephritis, with symptoms of fever and flank pain being common. Clinically, differentiating between the two can be difficult; however, management is significantly different as drainage of the abscess is necessary for treating pyonephrosis. While findings such as abdominal mass/fullness on the physical exam and pyuria can lead to higher clinical suspicion for the diagnosis, these findings are not specific to pyonephrosis [8]. Furthermore, 15% of patients may be asymptomatic [9]. Patients with pyonephrosis are sometimes misdiagnosed, leading to significant morbidity and mortality for the disease process. Common misdiagnoses such as ascites, cystic ovarian masses in females, mesenteric cysts, and cystic retroperitoneal tumors are reported to have led to erroneous trips to the operating room in 50% of cases of pyonephrosis, as reported by Gupta et al. [9].

Contrast enhanced CT imaging is the gold standard, but it is not readily available in most of the world. In addition to having a strong sensitivity and specificity, ultrasound is more readily available in most EDs due to its affordability. Using ultrasound can facilitate a quick diagnosis without the patient leaving the ED and can help determine the necessary therapeutic intervention for the disease process. The aim, with ultrasound and POCUS, is to differentiate simple hydronephrosis from pyonephrosis. While hydronephrosis appears as an anechoic collection within the collecting system, pyonephrosis is more echogenic and has a complex appearance, similar to other abscesses. The absence of such findings decreases the likelihood of pyonephrosis [10]. It is important to recognize the presence of pyonephrosis. While hydronephrosis in the setting of an infected stone can be life-threatening, it typically presents earlier in the disease process and does not cause the renal parenchyma destruction seen in cases of pyonephrosis, even in patients who have recovered [11].

Ureteric obstruction is the main cause of pyonephrosis, and stones are present in more than 70% of cases. The complete obstruction of the collecting system can be assessed by evaluating for ureteral jets in the bladder view with a sensitivity of 87% and a specificity of 96.4% [12]. Well-hydrated patients usually have 1-2 urinary jets per minute, with a good jet lasting at least 6 seconds [13]. For better results, the evaluation for urinary jet should be done over at least 10 minutes [14]. In this case, a ureteral jet was absent on the left side (Figure 3), causing a high suspicion for the complete obstruction of the collecting system.

The emergent drainage of the pus is a necessary step to treat pyonephrosis. Thus, in addition to developing the skills to make the appropriate diagnosis, physicians who work in low-resource settings where other specialists are not readily available must be capable of performing this life-saving intervention with ultrasound guidance.

## Conclusion

Pyonephrosis is usually a life-threatening condition due to the high risk of septic shock if it is not recognized and treated on an emergent basis. Internal or external ureteral blockage are the leading causes for the pathology. In resource-limited settings, timely and appropriate ultrasound diagnosis and decompression are the cornerstones of treatment. Without the appropriate diagnosis and intervention by an emergency physician in these settings, the mortality of these patients approaches 100%.
